# RAD51: Beyond the break

**DOI:** 10.1016/j.semcdb.2020.08.010

**Published:** 2021-05

**Authors:** Isabel E. Wassing, Fumiko Esashi

**Affiliations:** Sir William Dunn School of Pathology, University of Oxford, Oxford, UK

**Keywords:** RAD51, Double-stranded DNA breaks, Homologous recombination, Replicative stress, Fork protection

## Abstract

As the primary catalyst of homologous recombination (HR) in vertebrates, RAD51 has been extensively studied in the context of repair of double-stranded DNA breaks (DSBs). With recent advances in the understanding of RAD51 function extending beyond DSBs, the importance of RAD51 throughout DNA metabolism has become increasingly clear. Here we review the suggested roles of RAD51 beyond HR, specifically focusing on their interplay with DNA replication and the maintenance of genomic stability, in which RAD51 function emerges as a double-edged sword.

## 1 Introduction

Despite encoding the hereditary information that underlies all cellular processes, the DNA molecule is inherently unstable and readily acquires mutations which drive cellular malfunction and disease progression in vertebrates. Among the DNA lesions encountered by the cell, DNA double-stranded breaks (DSBs) are particularly lethal if left unrepaired and can give rise to chromosomal rearrangements if repaired erroneously [[1], [2], [3]]. As the main catalyst of homologous recombination (HR), one of the central mechanisms for DSB repair, the RAD51 recombinase has long been considered a major guardian of genomic stability. However, within the past decade, novel roles for RAD51 have been identified beyond this classical DSB repair function. Here we review these additional functions of RAD51 to re-evaluate its impact on genomic stability and cell survival.

## 2 RAD51 supports cell viability in vertebrates

Two-ended DSBs, such as those generated by ionising radiation, can be repaired by two major pathways: non-homologous end joining (NHEJ), which involves the direct ligation of the DNA ends to seal the break, and HR. HR utilises a homologous sequence as a template for DNA synthesis to replace the broken DNA, which, provided the identical sister chromatid is used, enables error-free repair of the break. RAD51 catalyses this process by promoting strand-invasion of a resected 3′ single-stranded DNA (ssDNA) end into the homologous repair template [4]. RAD51 has been extensively studied in the context of two-ended DSB repair, the *in vivo* efficiency of which is commonly assessed by functional assays which monitor the repair of enzymatically induced (two-ended) DSBs in reporter genes [5]. While HR can be understood to encompass a multitude of processes that involve extended ssDNA and homology-driven strand annealing, in this review, we utilise the term ‘HR’ to indicate any reaction that requires RAD51-mediated strand exchange at DNA breaks. Formation of the HR mediator complex, comprised of the breast cancer 1 (BRCA1), the breast cancer 2 (BRCA2) and the partner and localiser of BRCA2 (PALB2) proteins, plays a major role in promoting RAD51 recruitment to the break site, where BRCA2 facilitates assembly of the RAD51 nucleoprotein filament at the resected DNA end [[6], [7], [8]].

RAD51 orthologues are widely found throughout evolution, but its importance in cell survival varies between species. Deletion of the RAD51 homolog in budding yeast (*rad51*) or fission yeast (*rhp51*) confers high sensitivity to γ-irradiation but is not otherwise detrimental to cell viability, suggesting that the importance of Rad51/Rhp51 is specific to the repair of DSBs [9,10]. Conversely, RAD51 is generally thought to be essential for cell viability in vertebrates – RAD51 null mutation leads to embryonic lethality in mice, and triggers G2/M arrest followed by cell death in chicken lymphocyte DT40 cells [11,12]. Embryonic lethality is similarly reported in mice deficient for HR-mediator proteins BRCA1 or BRCA2, as well as NHEJ factors XRCC4 or DNA ligase IV (LIG4) [[13], [14], [15], [16]]. In the context of a multicellular and mitotically growing organism, where cells can constantly be replenished, cell death may be preferable to uncontrolled cell growth that leads to tumorigenesis. Indeed, the tumour suppressor p53 evolutionarily emerged in multicellular organisms and is a key factor to induce cell death upon DNA damage [17]. Significantly, while p53-deficiency rescues embryonic lethality in XRCC4^−/−^ or LIG4^-/-^ mice, this is not the case in BRCA1-, BRCA2- or RAD51-null mice [[18], [19], [20]], highlighting the importance of HR, relative to NHEJ, for vertebrate viability.

Interestingly, the contribution of HR in repairing two-ended DSBs is drastically reduced with increasing complexity of the organism – while *S. cerevisiae* predominantly employs HR for the repair of radiomimetic induced DSBs, NHEJ repairs at least 75 % of enzymatically induced breaks in immortalised human fibroblasts, and up to 85 % of X-ray induced DSBs in G2 in primary human fibroblasts [[21], [22], [23]]. Indeed, NHEJ has been characterised as a fast-acting repair mechanism in human cells, initially targeting all DSBs in G2, while HR is observed at those breaks where NHEJ cannot progress efficiently due to the presence of clustered DNA damage or surrounding heterochromatin [24]. Moreover, the NHEJ pathway, triggered by the association of abundant nuclear proteins Ku and DNA-dependent protein kinase catalytic subunit (DNA-PKcs), can compensate for the loss of HR at two-ended DSBs if end-resection is inhibited [24]. Altogether, two-ended DSB repair predominantly depends on NHEJ in human cells, such that the role of RAD51 in this process does not provide a satisfying explanation for the lethality of RAD51 deletion. Therefore, RAD51 seems to play an important role beyond two-ended DSB repair in vertebrates.

## 3 RAD51 is central to DNA replication under stress

### 3.1 RAD51 essentiality – the impact of genome size on replication stress and associated breakage

In an elegant study which identified genes that become essential upon the tetraploidisation of budding yeast, Rad51, as well as other players in the HR pathway, was required to sustain cell viability [25]. Given that replication-associated DNA damage was also increased in tetraploid yeast, this finding suggests that genome size and associated difficulties in replication dictate the importance of RAD51. Indeed, DNA metabolism itself can give rise to DSBs when the stable double-stranded helix is temporarily unwound and becomes vulnerable to nucleolytic attack. In particular, conditions of replication stress, in which replication slows down or stalls, can render DNA in this vulnerable state and hence represent a major endogenous source of DSBs. Vertebrates experience enhanced replication stress due to the increased size and complexity of the genome compared to yeast, such that the importance of RAD51 for cell survival may reflect an elevated level of endogenous DSBs arising during normal proliferation.

Importantly, fork breakage gives rise to one-ended DSBs that cannot be faithfully repaired *via* NHEJ, as no corresponding DNA end exists for religation. HR at such breaks prevents the toxic consequences of NHEJ, such as the formation of radial chromosomes. Indeed, the frequency of radial chromosomes in BRCA1-deficient cells decreases upon deletion of NHEJ players, and radial chromosomes generated upon the exposure to camptothecin (CPT), a topoisomerase I poison, were alleviated by DNA-PKcs inhibition and EXO1-mediated end-resection. [26,27]. The observed genomic instability resulting from NHEJ at endogenous or replication-dependent breaks suggests that RAD51-mediated repair is specifically important at one-ended DSBs. Indeed, RAD51 recruitment upon ionizing radiation (IR) is largely replication-dependent, suggesting that RAD51 primarily acts on a subset of DSBs that arise from replication fork breakage upon encountering IR-induced lesions on the template strand [28].

### 3.2 RAD51 in break-induced replication

One-ended DSB repair at a broken replication fork is of particular significance for cell survival as it can assist the resumption of replication, referred to as break-induced replication (BIR). As reviewed previously [29], detailed analysis in budding yeast has revealed that BIR is mediated by Rad51, but also takes place in the absence of Rad51. Rad51-independent BIR is proposed to be initiated by a Rad52-mediated strand annealing reaction [30,31]. Further studies in budding yeast established that, contrary to canonical chromosomal replication, BIR is a conservative DNA synthesis mechanism, whereby the invading DNA strand is displaced from the D-loop *via* branch migration to serve as a replication template for the lagging strand [32,33]. BIR in budding yeast can also be distinguished from canonical replication by its dependence on the ATP-dependent 5′-3′ DNA helicase Pif1 and the non-essential Pol32 component of replicative DNA polymerase δ [32,34]. It is worth noting that the aforementioned characterisation of BIR in yeast was achieved by studying the repair of two-ended DSBs generated by HO (homothallic switching) endonuclease, where only one side of the break is homologous to a donor site elsewhere in the genome, thereby directing repair towards BIR. A similar reporter system was used in human U2OS cells to confirm the existence of BIR, which appears to be primarily RAD51-dependent [35]. RAD51-dependent BIR has also been demonstrated in a cell-free system using *Xenopus laevis* egg extract [36]. However, a recent study revealed the involvement of RAD52 during BIR in U2OS cells upon replicative stress [37]. Analogous to BIR in yeast, this likely represents an alternative RAD51-independent mechanism of BIR, which relies on the strand annealing activity of RAD52.

Importantly, BIR-mediated replication restart does not necessarily protect against genomic instability. On the contrary, replication stress-induced BIR increases the frequency of copy-number alterations in human U2OS cells, whereas BIR at repetitive DNA accounts for gross chromosomal rearrangements and increased mutagenesis in fission and budding yeast, respectively [35,38,39]. Indeed, the cleavage of BIR intermediates by the crossover junction endonuclease MUS81, as well as the arrival of a converging fork, may be important to prevent long-tract BIR and its mutagenic consequences [40]. Furthermore, fork collapse upon extended hydroxyurea (HU) treatment in human U2OS cells was reported to inactivate replication entirely, such that its rescue relies on new origin firing [41]. Considering the mutagenic consequences of BIR, this likely represents a preferable solution to replication fork collapse than BIR-mediated fork restart.

### 3.3 RAD51 at stalled, but unbroken, replication forks

Aside from the proposed roles of RAD51 following replication fork collapse, accumulating evidence in recent years suggest that RAD51 also plays a role prior to fork breakage. Specifically, RAD51 promotes replication fork reversal, a process in which stalled replication forks are reversed and remodelled into a ‘chicken-foot’ structure. While fork reversal was first demonstrated in bacteriophage T4 [42], this research was recently extended to vertebrates, where fork reversal is recognised as a frequent response to fork uncoupling induced by a range of genotoxic reagents [43]. Several factors mediating fork reversal have been identified, including the SWI/SNF family translocases ZRANB3, SMARCAL1 and HLTF, as well as poly(ADP-ribose) polymerase 1 and RAD51 [[43], [44], [45], [46], [47]]. Surprisingly, fork reversal does not require the strand exchange activity of RAD51, which is critical for homologous recombination [48]. As reviewed previously [49], it is not currently fully understood how RAD51 promotes fork reversal, although it has been hypothesised that this could involve RAD51 binding to and hence stabilising the reversed arm of the fork. Indeed, RAD51 has been shown to prevent restoration of the ‘normal’ fork structure *in vitro* [50].

#### 3.3.1 RAD51 promotes genomic stability

**Fig. 1:**
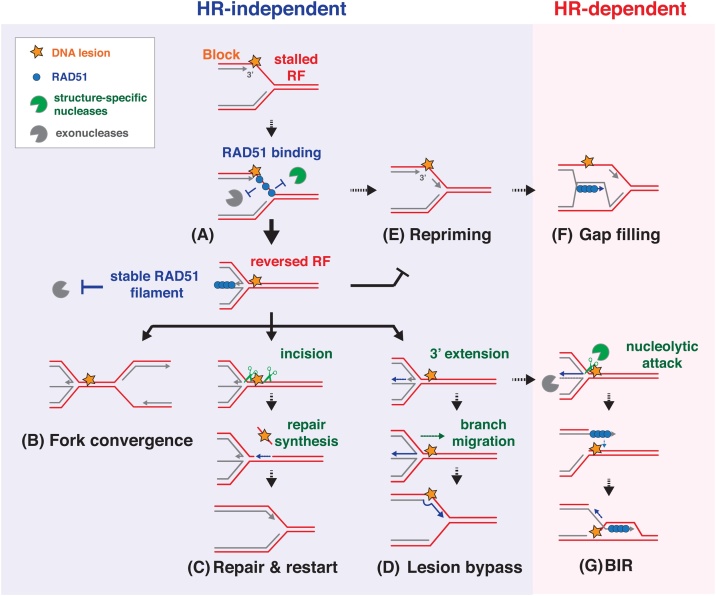
**Roles of RAD51 at stressed replication forks.** Simplified depiction of the mechanisms by which RAD51 protects the stalled replication fork (RF). RAD51 recruitment to replication forks mediates fork reversal, as well as fork protection from breakage, either from mechanical force or nucleolytic activity (A). This initial process is independent of long RAD51 filament formation [51,80], although the detailed mechanisms remain elusive. Subsequently, RAD51 forms a stable nucleoprotein filament on the regressed arm of the ‘chicken foot’ structure to protect the newly formed DNA end from nucleolytic attack, for example by MRE11. This allows for the stabilisation of the stalled fork until fork convergence (B) or removal of the replication-obstructing lesion ahead of the fork (**C**). The regressed fork also offers the opportunity for 3′ end extension using the opposite strand as template, hence allowing for lesion bypass (**D**). Fork reversal prevents excessive repriming of DNA synthesis (E), which leads to the accumulation of ssDNA behind the fork. Remaining ssDNA can be repaired by RAD51-mediated template-switching, followed by gap-filling (**F**). In the absence of RAD51 stabilisation at the regressed arm the reversed fork may be targeted by MUS81 or other structure-specific nucleases, leading to fork breakage followed by break-induced replication (BIR). Strand-invasion past the replication-obstructing lesion could be enabled by 3′ extension at the reversed fork, as depicted, or alternatively rely on (partial) homology ahead of the fork (**G**).

There are various proposed mechanisms by which fork reversal might aid genomic stability. Foremost, several studies have found that fork reversal prevents DSB formation, suggesting that the reversed fork represents a stable intermediate structure which is resistant to nucleolytic attack [43,45,47]. Indeed, RAD51 depletion severely reduces fork reversal and increases DSB formation under conditions of replication stress, suggesting that RAD51-mediated fork reversal protects the replication fork from breakage and hence contributes to genomic stability [43]. RAD51 is initially recruited to stalled replication forks independently of BRCA2 (discussed in section 3.5), and its association with the extended ssDNA, without forming a long RAD51 nucleoprotein filament, appears sufficient to initiate fork reversal [51] (Fig. 1A). Fork protection is enhanced upon the stabilisation of the RAD51 filament by BRCA2, which was shown to prevent nucleolytic degradation of nascent DNA at stalled replication forks [52]. BRCA1, FANCD2 and BOD1L similarly protect the replication fork through the stabilisation of the RAD51 nucleoprotein filament, thereby preventing replication stress-induced DSB formation [[53], [54], [55], [56]]. Given the mutagenic nature of either BIR or NHEJ mechanisms acting at broken replication forks, fork reversal may be an important strategy to maintain genomic stability until replication is rescued by a converging fork (Fig. 1B). Alternatively, fork reversal places the DNA lesion further away from the fork, where the double-stranded DNA context may promote its removal by conventional repair pathways, such as nucleotide excision repair, thereby enabling replication progression once the lesion has been repaired (Fig. 1C). Fork reversal similarly promotes interstrand crosslink (ICL) repair, which classically involves dual incisions on either side of the ICL in order to ‘unhook’ the lesion. Indeed, it has been shown that RAD51 is recruited to ICL-stalled forks at early stages in the repair process, and that fork reversal is required for XPF-ERCC1-dependent ICL unhooking [57,58]. This is in line with a role for RAD51-mediated fork reversal in promoting ICL excision. In contrast to other replication-blocking lesions, ICLs prevent separation of the double-stranded DNA helix and were long thought to block replication permanently unless the lesion is excised from the DNA. Intriguingly, recent reports suggest that the replication fork can continue past the obstructing ICL without requiring its removal. While the exact mechanism by which ICL traverse occurs is not yet clear, it is reportedly promoted by FANCM translocase activity as well as fork reversal [59,60]. Fork reversal has also been proposed to directly promote progression of the stalled fork *via* a template-switching mechanism. As the stalled DNA strand pairs with the sister chromatid upon fork reversal, this provides an undamaged replication template with which to bypass the replication-obstructing DNA lesion (Fig. 1D). Replication restart by template switching was observed to occur *via* a regressed fork intermediate in bacteriophage T4, and *in vitro* reconstitution indicated that RAD51 can indeed promote DNA lesion bypass in this manner [42,50]. Intriguingly, while the reversed replication fork may therefore serve as an important intermediate to promote the controlled progression past a lesion, DNA fiber experiments indicate that fork reversal induces an overall slowing of replication [43,45,47]. A detailed study of fork progression upon ICL induction revealed that fork reversal is not limited to those forks that encounter the lesions, but also occurs at unchallenged forks [59]. Fork reversal thus seems to function as a general fork slowing mechanism which prevents fork-lesion collision, thereby promoting repair of such lesions away from the replication fork [59].

Fork reversal has additionally been proposed to prevent replication restart by repriming. PRIMPOL, a translesion synthesis (TLS) polymerase with primase activity in vertebrates, has been shown to mediate replication restart at stalled forks by repriming DNA replication downstream of UV- or cisplatin-induced bulky lesions [61,62]. PRIMPOL-mediated repriming is favoured in the absence of proficient fork reversal, suggesting that fork reversal may normally prevent this PRIMPOL-mediated replication restart [62] (Fig. 1E). It is not yet understood how the pathway choice between fork reversal or repriming is regulated in response to fork stalling. However, reprimed replication leads to the accumulation of ssDNA gaps behind the fork which, unless efficiently filled in, may be processed into DSBs and hence represent a source of potential genomic instability [62,63].

Separate from its role in fork reversal, RAD51 has also been proposed to limit the accumulation of ssDNA gaps behind the fork by HR-mediated post-replicative gap filling (Fig. 1F). Studies in budding yeast have implicated Rad51 in the post-replicative repair of ssDNA gaps *via* a template switch mechanism behind the fork [64,65]. Furthermore, through the use of a thermosensitive degron system, RAD51 depletion in DT40 cells was shown to cause the accumulation of ssDNA structures throughout S phase, culminating in G2 arrest [66]. Interestingly, the complementation of RAD51 in G2, but not S-phase, led to the reduction of these ssDNA structures, consistent with a G2-specific post-replicative role for RAD51-mediated gap filling. In line with this, RAD51 depletion in *Xenopus* egg extract led to the accumulation of ssDNA gaps behind the fork [67]. Alternatively, analogous to the role of RAD51 at stalled replication forks in protecting nascent DNA from nucleolytic attack [52], these observations could also indicate a protective role for RAD51 at small ssDNA gaps and nicks behind the fork. Lastly, a recent study using *Xenopus* egg extract also demonstrated that RAD51 directly interacts with the Pol α-primase complex and promotes the association of replicative DNA polymerases Pol α and Pol δ with chromatin. Given that RAD51 depletion leads to the accumulation of ssDNA both at and behind the fork, this introduces the intriguing possibility that RAD51 stabilises the replisome to promote continued DNA synthesis at stalled or reversed forks [67,68].

#### 3.3.2 RAD51 as a potential source of genomic instability

As discussed above, emerging evidence indicates that the stabilisation of the RAD51 nucleoprotein filament plays a crucial role in the protection of the stalled replication forks against nucleolytic degradation. Interestingly, several recent studies suggest that the role of RAD51 in fork protection is particularly important after fork reversal; inhibition of fork reversal rescues the nascent DNA degradation otherwise observed at stalled forks in a BRCA1- or BRCA2-deficient background [53,[68], [69], [70]]. This suggests that, paradoxically, fork reversal provides the entry point for nucleolytic DNA degradation at stalled forks: RAD51-mediated fork reversal can, in the absence of further stabilisation of RAD51 by BRCA1 or BRCA2 at the regressed arm, promote fork degradation and genome instability. Indeed, uncontrolled processing of the reversed fork arm was also shown to generate a substrate for MUS81-dependent cleavage, such that the lack of RAD51 stabilisation at reversed forks culminates in fork breakage [70]. The resultant broken forks subsequently engage in BIR (Fig. 1G), which may potentially start at regions containing partial homology or repeats. Therefore, rather than protecting against genomic instability, RAD51-mediated fork reversal can trigger mutagenic replication in BRCA-deficient cancer cells, suggesting that the reversed fork is an inherently vulnerable DNA structure. Interestingly, excessive fork reversal activity of SMARCAL1 reportedly induces nascent DNA degradation and DNA breakage at stalled forks, such that DNA damage at reversed forks is not necessarily limited to conditions in which fork protection is compromised [71]. Indeed, the importance of regulating fork reversal is highlighted by the discovery of RADX, which antagonises RAD51 bound at reversed forks to prevent excessive fork reversal and associated fork breakage [72].

Despite the potential threats outlined above, fork breakage may be beneficial under certain conditions. While BIR is indeed mutagenic, a limited incidence of break-induced fork restart may assist the completion of genome replication when cells are exposed to mild replicative stress. In this case, BIR prevents chromosome missegregation that would otherwise result from the mitotic division of an under-replicated genome. Indeed, ATAD5, a PCNA unloader which forms the alternative pentameric replication factor C (RFC)-like complex (RLC), was shown to stimulate replication restart in response to hydroxyurea (HU) by promoting RAD51-mediated fork reversal and subsequent fork breakage [73]. Interestingly, ATAD5 haploinsufficiency in mice increases HU-induced micronuclei formation, a hallmark of aberrant chromosome segregation [73]. Therefore, while RAD51-generated reversed fork structures may be inherently susceptible to nucleolytic attack and subsequent fork breakage, it is precisely this fork instability that enables the engagement of BIR to alleviate genomic instability upon replicative stress. Furthermore, controlled resection or branch migration at the reversed fork enables the restoration of a functional replication fork, such that the excessive stability of the reversed fork may hamper the resumption of replication [74,75]. Both fork reversal and the timely dismantling of this structure are therefore crucial to safeguard replication progression and genomic stability in conditions of replication stress. Whether RAD51 at stalled forks ultimately maintains or disturbs genomic stability likely depends on the nature and the severity of the genotoxic stress, the regulation of RAD51 activity at the fork and the nature of the surrounding DNA sequence. Indeed, repetitive regions may elicit illegitimate template switching events, possibly in a RAD51-dependent manner, culminating in chromosomal rearrangements [76,77].

### 3.4 What is more important, repair or protection?

Considering the myriad functions for RAD51 at stalled or broken replication forks, RAD51 and replication appear inextricably linked. Given the fact that neither RAD51-mediated fork protection nor fork reversal requires RAD51 strand exchange activity, it is tempting to speculate that the role of RAD51 in HR is not of great importance to cell viability [48,52]. Indeed, expression of the RAD51 K133R mutant, which, although competent in strand exchange *in vitro* [78], is defective in HR *in vivo* [5], rescues cell viability in RAD51-null DT40 cells, suggesting that HR is dispensable when fork protection is sustained [78]. Furthermore, the loss of BRCA2-mediated fork protection was shown to underlie the lethality of BRCA2 deficiency in embryonic stem cells [79]. Conversely, the Fanconi anaemia-associated dominant-negative RAD51 T131P mutation negatively impacts the stability of the RAD51 nucleoprotein filament, thereby abrogating replication fork protection, while maintaining HR proficiency [51,80]. Nonetheless, expression of RAD51 T131P mutant does not abrogate cell survival, suggesting that the loss of RAD51-mediated fork protection can be tolerated in the presence of its HR activity or that the residual functions of T131P are incompletely understood. Notably, a recent study found that BRCA2-mediated fork protection is dispensable for the viability of untransformed human cells [81]. Indeed, BRCA2 was found to prevent under-replication in a way that depends on its interaction with RAD51, but not on its role in fork protection. This study therefore suggests that BRCA2 and RAD51 facilitate efficient replication in untransformed cells, likely through HR, although a RAD51-mediated role unrelated to canonical HR and fork protection may deserve consideration. Overall, it appears that RAD51-mediated fork protection ensures cell viability in the absence of HR activity, while HR is essential to support cell viability upon the loss of RAD51-mediated fork protection.

### 3.5 Mechanisms recruiting RAD51 to the replication fork

**Fig. 2:**
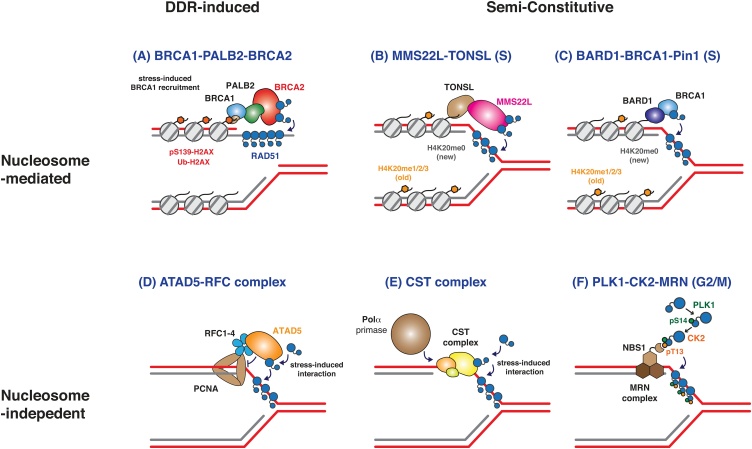
**Mechanisms promoting RAD51 recruitment.** Simplified depiction of mechanisms that promote RAD51 recruitment to sites of DSBs (A) and stalled replication forks (B–E). RAD51 recruitment to sites of DSBs is largely mediated by the BRCA1-PALB2-BRCA2 complex, which is initiated by BRCA1 recruitment to DNA damage markers, such as S130-phosphorylated and/or ubiquitinated H2AX (A). RAD51 recruitment at stalled replication forks may be mediated by the MMS22L-TONSL complex (B) or the BARD1-BRCA1 complex (**C**), both of which bind the histone H4 non-methylated at K20 (H4K20me0) that is present in newly synthesised DNA. RAD51 directly binds MMS22 L and a Pin1-generated BRCA1 isomer, providing a constitutive RAD51 loading mechanism in S-phase when H4K20 remains unmethylated. Upon replicative stress the ATAD5-RFC complex is recruited to replication forks, where it removes PCNA and facilitates RAD51 loading to replication forks (D). The CST complex, an RPA-like complex composed of CTC1, STN1 and TEN1, is enriched at telomeres and GC-rich repeats and recruits DNA polymerase Pol α-primase. Replicative stress triggers its interaction with RAD51, promoting the loading of RAD51 (**E**). RAD51 S14 is targeted by PLK1 transiently after DNA damage or during late G2-M. This is followed by T13 phosphorylation by CK2, which creates a binding motif for the NBS1 FHA domain of the MRE11-RAD50-NBS1 complex, which travels together with the replication machinery. The direct interaction of T13-phosphorylated RAD51 with NBS1 hence mediates RAD51 recruitment to stalled forks (**F**).

While the protective function of BRCA2 at reversed replication forks is well-described, the persistence of RAD51-mediated fork reversal in BRCA2-deficient cells suggests that RAD51 recruitment to the stalled fork can be mediated independently of BRCA2. Indeed, the function of BRCA2 in fork protection was shown to be independent of its role in HR (*i.e.* RAD51 recruitment to DSBs – see Fig. 2A) [52]. Furthermore, replication stress-induced RAD51 recruitment remains intact in BRCA2-deficient cancer cells and BRCA2-hypomorphic mutant embryonic stem cells [79,82]. Together, these observations suggest that BRCA2 is dispensable for RAD51 recruitment at stalled forks. How then is RAD51 recruitment to the stalled fork achieved?

#### 3.5.1 Constitutive association of RAD51 loaders

One way in which factors can be directed towards replicated DNA, including at stalled replication forks, is *via* their interaction with newly incorporated nucleosomes containing histone H4 non-methylated at K20 (H4K20me0). This applies to the MMS2L-TONSL complex, which constitutively binds H4K20me0, and is involved in RAD51 recruitment to stalled replication forks (Fig. 2B) [83]. The depletion of MMS22L-TONSL reduces DNA damage-induced RAD51 foci, but not BRCA2 foci, suggesting that MMS22L-TONSL recruits RAD51 in a BRCA2-independent manner [84]. Interestingly, MMS22L-depleted cells are sensitive to CPT-induced replication stress, but not to IR, further suggesting that MMS22L-TONSL is specifically important for RAD51 recruitment to stalled replication forks [84]. Indeed, the MMS22L-TONSL complex localises to stalled replication forks and MMS22L was found to promote reversal of uncoupled forks [85,86]. However, *in vitro* analysis showed that MMS22L-TONSL is not able to mediate RAD51-dependent strand exchange in the presence of RPA, such that MMS22L-TONSL-dependent loading of RAD51 at the fork presumably requires the collaborative action of other factors for the removal of RPA [85]. More recently, the BARD1-BRCA1 complex was also reported to associate with H4K20me0-containing nucleosomes on newly replicated DNA, in a manner that is important for the protection of replication forks [87] (Fig. 2C). Furthermore, the conformational alteration of BRCA1 by the prolyl isomerase Pin1 has been shown to trigger its direct interaction with RAD51, such that it protects replication forks independently of BRCA2 [56]. Since the H4K20me0 nucleosome serves as an immediate marker of nascent DNA, the above-described mechanisms allow for the prompt loading of RAD51 when replication forks encounter obstacles.

#### 3.5.2 Replication stress-induced RAD51 loaders

ATAD5 is another factor that directly binds RAD51 and promotes its recruitment to stalled forks (Fig. 2D). Unlike the aforementioned H4K20me0 binding factors, ATAD5 acts in response to replicative stress. Interestingly, ATAD5-mediated recruitment of RAD51 depends on the PCNA unloading activity of ATAD5 at stalled forks [73]. It is not yet clear whether ATAD5-mediated RAD51 recruitment promotes fork reversal or fork protection. Nonetheless, since PCNA was found to inhibit fork reversal *in vitro*, this observation may suggest that RAD51 recruitment at stalled forks requires prior fork reversal and, therefore, ATAD5-mediated PCNA removal [73]. This is in line with the idea that RAD51 promotes fork reversal by binding to and stabilising the regressed arm of the chicken foot structure [49]. Additionally, the CST complex, composed of CTC1, STN1 and TEN1, has been shown to act as a loader of RAD51 at telomeres and other genomic loci susceptible to replication stress [88,89]. The CST complex is an RPA-like complex which is best characterised with regard to its role in telomere maintenance, where it bridges the shelterin complex and Pol α to promote ssDNA fill-in synthesis [[90], [91], [92], [93]]. The CST complex is enriched at GC-rich repetitive regions genome-wide and, upon replicative stress, recruits RAD51 to these regions through direct interaction (Fig. 2E) [88]. Curiously, however, the CST complex was found to counteract IR-induced RAD51 recruitment in the absence of BRCA1 [94], suggesting a context-dependent role of this complex in RAD51 loading.

#### 3.5.3 Post-translational regulation of RAD51 for loading

Lastly, the sequential phosphorylation of RAD51 by Polo-like kinase 1 (PLK1) and casein kinase 2 (CK2) was shown to mediate RAD51 recruitment to stalled replication forks independently of BRCA1-PALB2-BRCA2 complex formation [[95], [96], [97]]. PLK1- and CK2-dependent phosphorylation of RAD51 mediates a direct interaction between RAD51 and the NBS1 component of the MRN (MRE11-RAD50-NBS1) complex, which acts as one of the earliest DSB sensors [95,98] (Fig. 2F). In this way, the PLK1-dependent phosphorylation of RAD51 assists its accumulation at sites of DNA damage and promotes HR even in a BRCA2-deficient background [95,96]. Since PLK1 is a mitotic kinase, its phosphorylation of RAD51 is cell cycle-regulated and peaks in late G2 phase and mitosis during an unperturbed cell cycle, introducing the possibility that RAD51 may also target DNA damage or stress during mitosis. Indeed, the resolution of under-replicated DNA during mitosis has been a focus of recent research [99,100].

## 4 RAD51 at difficult-to-replicate regions

**Fig. 3:**
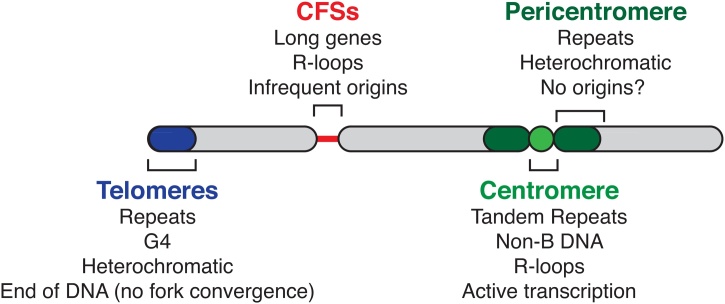
**Overview of difficult-to-replicate regions.** Simplified depiction of chromosomal regions that are ‘difficult-to-replicate’ and vulnerable to replicative stress. Proposed sources of replicative stress are also indicated. Common fragile sites (CFSs) often associate with long genes where stable association of the transcriptional machinery and RNA-DNA hybrids (R-loops) are found. Sparse replication origins within these genes also contribute to their vulnerability. Telomeres are composed of a GC-rich repetitive sequence, and G4 DNA structures and heterochromatic sub-telomeric regions exacerbate the challenge in replicating these regions. In the absence of a distal origin, fork convergence cannot overcome replication stalling. Centromeres and pericentromeric regions, spanning millions of base pairs in mammalian genomes, also comprise repetitive sequences. Non-B form DNA structures, R-loops and active transcription are found at centromeres, while pericentromeres are largely heterochromatic. The presence of origins within these regions remains unclear.

In vertebrates, numerous chromosomal regions have been identified inherently ‘difficult-to-replicate’, such that they may remain under-replicated when cells enter mitosis, particularly under conditions of mild replicative stress. Such regions include common fragile sites (CFSs), telomeres and centromeres (Fig. 3).

### 4.1 RAD51 at CFSs

CFSs are identified by their propensity to form metaphase chromosome ‘gaps’ upon exposure to mild replication stress [101,102], and generally coincide at long, transcriptionally active genes where replication-transcription collisions underlie the observed fragility of CFSs [103]. These replication stress-induced gaps do not necessarily indicate chromosomal breaks, but may also represent regions of the metaphase chromosome that are not condensed and are actively engaged in DNA synthesis, as indicated by the incorporation of the thymidine analog 5-Ethynyl-2′-deoxyuridine (EdU) in mitosis at these sites [100]. Careful cell synchronisation prior to EdU incubation confirms that DNA synthesis occurs during early mitosis and that this process is specifically initiated in mitosis [100]. A BIR-like mechanism has been proposed in which MUS81-mediated fork breakage at stalled replication intermediates triggers RAD52- and POLD3-dependent, but RAD51-independent, mitotic DNA synthesis (MiDAS) [100]. Indeed, the majority of MiDAS in U2OS cells was initially found to be conservative DNA synthesis, a key characteristic of BIR [104]. POLD3 is the human orthologue of budding yeast Pol32, the non-essential component of replicative DNA polymerase δ. Given that Pol32 is essential for BIR in budding yeast, but not for canonical S-phase replication, the POLD3-dependent nature of MiDAS supports the proposed BIR-like mechanism of MiDAS [34]. However, the POLD3-dependent nature of MiDAS does not exclude the possibility of a semi-conservative mechanism of DNA synthesis. Indeed, recombination-initiated replication re-start at a protein-induced replication fork barrier in fission yeast reportedly occurs by a semi-conservative, albeit non-canonical mechanism of DNA synthesis, where both leading- and lagging-strand are synthesised by Pol δ [105]. Furthermore, a recent study found that MiDAS events in U2OS cells were primarily semi-conservative in nature, suggesting that replication progression in mitosis could not be explained solely *via* the canonical BIR mechanism [106]. In this study, the authors propose a mechanism in which the broken replication fork is restored to a ‘normal’ replication fork through RAD52-dependent strand annealing and LIG4/XRCC4-mediated ligation [106]. It remains to be elucidated whether continued progression of replication forks in mitosis, induced without fork breakage, also accounts for the semi-conservative MiDAS observed, and whether RAD51 plays a role in this process. Indeed, given the differing reports to date, it is likely that diverse mechanisms underlie MiDAS depending on the specific genomic loci or replication conflicts in question. Importantly, since mitosis is the phase of the cell cycle during which the activities of various structure-specific nucleases, such as MUS81, are highest, it remains an open question how the active replication forks and/or DNA repair intermediates that arise during MiDAS are protected from nucleolytic cleavage [[107], [108], [109]].

### 4.2 RAD51 at telomeres

Telomeres have been also shown to undergo replication stress-induced MiDAS. Telomeric MiDAS similarly occurs *via* a conservative DNA synthesis that is dependent on RAD52, but not RAD51 [[110], [111], [112]]. While the majority of cancer cells maintain telomere length by the reverse transcriptase activity of the telomerase complex, 10–15 % of human cancers lack telomerase activity and rely on the homology-directed alternative lengthening of telomeres (ALT) pathway for telomere length maintenance [113]. Importantly, RAD52 promotes non-S-phase telomeric DNA synthesis specifically in ALT-positive cancer cell lines, and RAD52 deletion was shown to reduce telomere length in these cells [114,115]. It is therefore suggested that RAD52 plays a major role in ALT, and that telomeric MiDAS represents this telomere lengthening mechanism [112,114]. While these studies highlight the importance of RAD52 in this process, RAD51 has also been implicated in ALT. Specifically, the induction of telomeric DSBs stimulates inter-chromosomal clustering of telomeres in ALT-positive cell lines in a RAD51-dependent manner [116]. This damage-induced mobility of the telomere was proposed to mediate inter-chromosomal homology search preceding recombination between non-sister telomeres, analogous to the pairing of homologues in meiosis I. Indeed, telomere clustering in ALT-associated promyelocytic leukaemia bodies (APBs) is a hallmark of ALT, and APB formation has been linked to ALT activity [117,118]. Telomere clustering is thought to promote ALT by bringing telomeres together with a variety of DNA damage factors that are enriched at APBs, such as RAD51 and the BLM helicase [112,117,119]. Interestingly, BLM overexpression in the ALT-positive U2OS cell line increases telomere extension in a RAD51- and POLD3-dependent manner, suggesting that RAD51-dependent BIR can indeed underlie ALT [120]. Nonetheless, both the role of RAD51 and the importance of APBs in ALT remain contentious. One study showed that, while RAD51 depletion reduces DSB-induced telomere clustering, it did not significantly reduce telomere lengthening in ALT positive cells, arguing that RAD51-dependent telomere clustering is not essential for ALT [115]. To further complicate matters, endogenous telomeric DNA synthesis was shown to occur almost exclusively at APBs in G2, in a manner dependent on BLM and POLD3/4, but not on RAD51 [121]. It is important to highlight, however, that ALT has been studied under a wide range of experimental conditions – while some look at endogenous ALT, others induce ALT using exogenous stresses or protein overexpression, or focus on a specific phase of the cell cycle. These variations likely account for the conflicting reports of RAD51 involvement in ALT. Indeed, RAD51 recruitment to the telomere was recently shown to be promoted by endonuclease-induced telomeric DSBs, but not upon ROS-induced telomeric DNA damage [122]. Taking everything into consideration, it is clear that RAD52 plays an important role in ALT, analogous to the Rad51-independent (Type II) ALT mechanism that has been described in telomerase-deficient budding yeast [123]. Importantly, ALT also occurs *via* a Rad51-dependent (Type I) mechanism in budding yeast, reinforcing the idea that a subset of telomeres in human cells may similarly undergo ALT in a RAD51-dependent manner. Future research will be needed to clarify the molecular mechanisms of ALT and their regulation in human cells.

## 5 Concluding remarks

Dedicated research over the past decade has elucidated the functions of RAD51 beyond two-ended DSB repair. While the importance of RAD51 in replication has specifically emerged, this is clearly a double-edged sword, which can both prevent and induce genomic instability depending on the circumstances. Overall, a delicate balance of factors stabilising and antagonising RAD51 appear to coexist at stalled forks. Exactly how the optimal combination of these opposing activities is achieved will no doubt be the focus of future investigations. The function of RAD51 at specific genomic loci also merits further investigation. For example, the centromere represents another genomic region which is inherently difficult-to-replicate. As the platform for kinetochore assembly, it is essential for accurate chromosome segregation and the maintenance of genomic stability. Therefore, to fully understand the impact of RAD51 on genomic stability, it will be important to investigate the role of RAD51 at the centromere, which is currently understudied. It is also noteworthy that the essentiality of RAD51 varies even between different vertebrate species. For example, while RAD51 null mutants are embryonically lethal in mice, RAD51-null zebrafish survive into adulthood [124]. It has been proposed that the presence of maternally-derived RAD51 contributes to the viability of RAD51 null mutants in zebrafish. If this is indeed the case, it is possible that, at least in some organisms, the essentiality of RAD51 may be specific to the early stages of embryogenesis. Does genome size, or a fundamental difference in DNA metabolism underlie the varied importance of RAD51 between organisms? It appears that we are still far from a complete understanding of the mechanisms by which RAD51 ensures viability, a critical question to be asked in future studies.
